# A paradigmatic case of haemolysis and pseudohyperkalemia in blood gas analysis

**DOI:** 10.11613/BM.2019.011003

**Published:** 2019-02-15

**Authors:** Gian Luca Salvagno, Davide Demonte, Giuseppe Lippi

**Affiliations:** Section of Clinical Biochemistry, University of Verona, Verona, Italy

**Keywords:** potassium, hyperkalemia, preanalytical variability, diagnostic errors

## Abstract

A 51-year old male patient was admitted to the hospital with acute dyspnea and history of chronic asthma. Venous blood was drawn into a 3.0 mL heparinized syringe and delivered to the laboratory for blood gas analysis (GEM Premier 4000, Instrumentation Laboratory), which revealed high potassium value (5.2 mmol/L; reference range on whole blood, 3.5-4.5 mmol/L). This result was unexpected, so that a second venous blood sample was immediately drawn by direct venipuncture into a 3.5 mL lithium-heparin blood tube, and delivered to the laboratory for repeating potassium testing on Cobas 8000 (Roche Diagnostics). The analysis revealed normal plasma potassium (4.6 mmol/L; reference range in plasma, 3.5-5.0 mmol/L) and haemolysis index (5; 0.05 g/L). Due to suspicion of spurious haemolysis, heparinized blood was transferred from syringe into a plastic tube and centrifuged. Potassium and haemolysis index were then measured in this heparinized plasma, confirming high haemolysis index (50; 0.5 g/L) and pseudohyperkalemia (5.5 mmol/L). Investigation of this case revealed that spurious haemolysis was attributable to syringe delivery in direct ice contact for ~15 min. This case emphasizes the importance of avoiding sample transportation in ice and the need of developing point of care analysers equipped with interference indices assessment.

## Introduction

Hyperkalemia is conventionally defined as a serum or plasma potassium concentration > 5.5 mmol/L or > 5.0 mmol/L, respectively. Albeit the prevalence of this electrolyte disturbance is mostly unknown in the general population, it was found to be as high as 10% in hospitalized patients (*1*). Hyperkalemia, which may be associated with severe complications such as fasciculation, paresthesia and arrhythmias, can be due to either biological causes (*e.g.*, renal failure, increased potassium intake, administration of certain drugs such as β-blockers, digoxin and potassium sparing diuretics, among others), or to a vast array of preanalytical problems such as blood sample haemolysis, prolonged tourniquet placement, fist clenching, sample contamination from infusive routes, prolonged storage of uncentrifuged blood, as well as by leukocytosis or thrombocytosis (*1*-*4*). Among the various causes of pseudohyperkalemia (also known as spurious hyperkalemia), haemolysis is indeed the more frequent and may hence be accompanied by a wrong diagnosis of hyperkalemia and an ensuing inappropriate clinical decision making (*e.g.*, avoidable emergency referral and/or unjustified lowering of an otherwise normal *in vivo* potassium concentration) (*5*). We describe here a paradigmatic case of pseudohyperkalemia in blood gas analysis due to inappropriate transport conditions of a heparinized syringe. A written informed consent for publication of this case report was obtained directly from the patient.

## Case report

A 51-year old male patient with a chronic history of asthma managed with β2 adrenergic receptor agonists (salbutamol) was admitted to the asthma clinic at the University hospital of Verona (Italy) with wheezing, coughing, whistling sounds during breathing and worsening dyspnea, lasting for approximately 24 hours. The physical examination revealed wheezing during normal breathing, prolonged phases of forced exhalation and use of accessory muscles for breathing. As commonplace in these cases, the physician immediately prescribed a blood gas analysis for investigating the effectiveness of gas exchange and state of voluntary respiratory control. Venous rather than arterial blood was hence initially drawn by a nurse into a 3.0 mL heparinized syringe (Smiths Medical, Minneapolis, MI, United States), and delivered by hand to the central laboratory, for analysis of blood gases.

## Laboratory analyses

**Figure 1:**
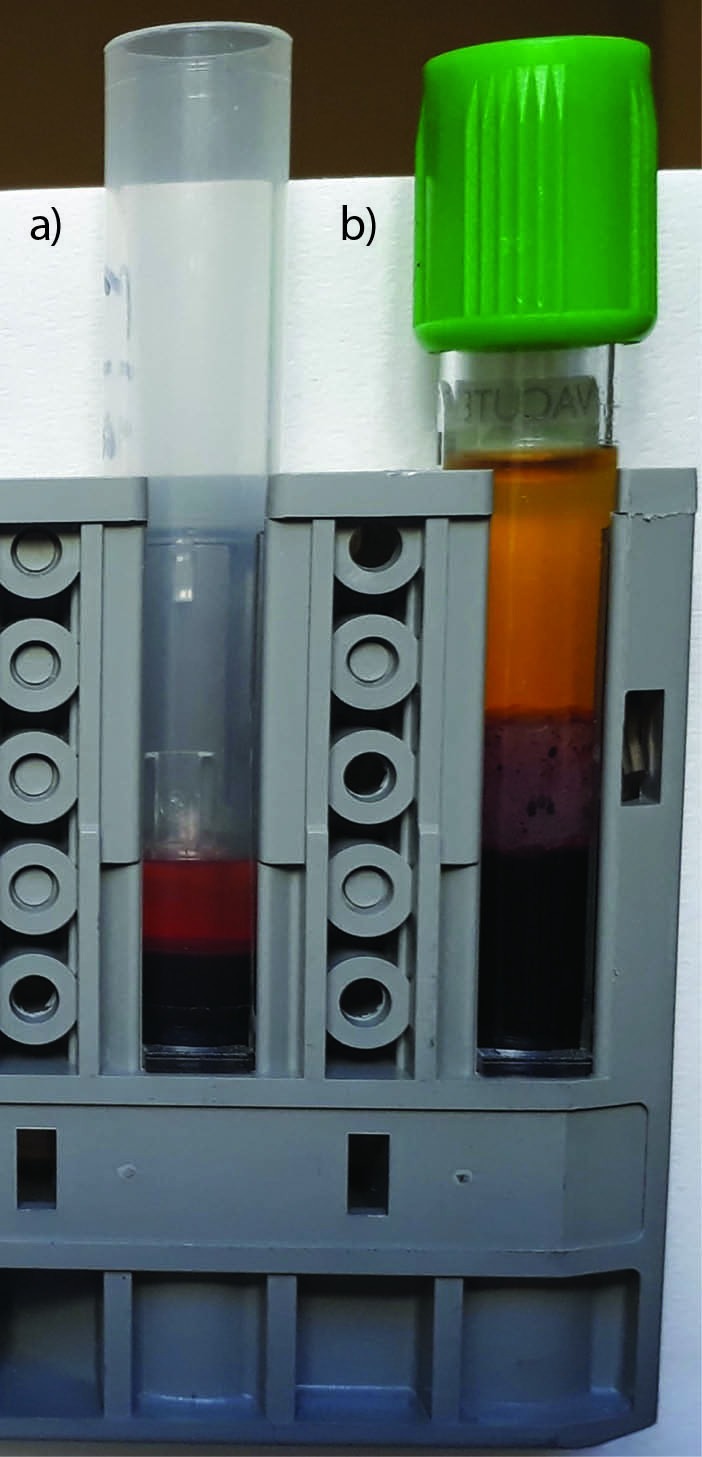
Visual appearance of heparinized blood after centrifugation. (a) heparinized syringe for blood gas analysis; (b) standard lithium-heparin blood tube.

The blood gas analysis, performed with GEM Premier 4000 (Instrumentation Laboratory, Bedford, MA, United States), revealed decreased values of partial pressure of oxygen (pO_2_, 16 mmHg; reference range, 30 - 40 mmHg) and oxygen saturation (SO_2_, 38%; reference range, > 75%), a slightly increased partial pressure of carbon dioxide (pCO_2_, 57 mmHg; reference range, 40 - 50 mmHg) and high value of potassium (*i.e.*, 5.2 mmol/L; local reference range on whole blood, 3.5 - 4.5 mmol/L). No alarms were generated by the analyser during sample testing. The potassium value was unexpected by the requesting physicians, so that a second venous blood sample was immediately drawn by direct venipuncture into a 3.5 mL lithium-heparin blood tube (Vacutest, Kima, Padova, Italy), and sent to the local laboratory for repeating potassium testing. The sample was separated by centrifugation (1500xg for 10 min at room temperature) upon delivery, and the analysis performed on Cobas 8000 (Roche Diagnostics, Risch-Rotkreuz, Switzerland) revealed normal values of plasma potassium (*i.e.*, 4.6 mmol/L; local reference range in plasma, 3.5 - 5.0 mmol/L) and haemolysis index (*i.e.*, 5; 0.05 g/L). Due to the suspicion of spurious haemolysis in the previous blood gas specimen, heparinized blood was transferred from the syringe into a plastic tube and plasma was separated by centrifugation (1500xg for 10 min at room temperature). Both potassium and haemolysis index were then measured in the syringe heparinized plasma, using Cobas 8000 analyser. The visual analysis of plasma after centrifugation was already consistent with the presence of haemolysis (*i.e.*, mildly red hue) (Figure 1), which was then confirmed by an increased value of haemolysis index (*i.e*., 50; 0.5 g/L). The potassium value in the centrifuged heparinized plasma confirmed the earlier finding of pseudohyperkalemia (*i.e.*, 5.5 mmol/L). The patient had already been treated with a dose of aerosol beclometasone dipropionate, which was effective to alleviate the symptoms at the time of receiving test results of the second sample, so that a second blood gas analysis was deemed unnecessary. The patient was finally discharged with a prescription for inhaled salbutamol and beclometasone dipropionate to be used during acute exacerbation of asthma.

## What happened?

A deep investigation of this case revealed that the blood gas syringe was transported to the local laboratory in direct contract with ice. In fact, in order to accelerate the transport of the specimen, the nurse positioned the blood gas syringe into a metal try, and the syringe was entirely submerged by the ice contained into the tray (rather than using a coolant) for approximately 15 min before being removed by the laboratory staff upon arrival in the laboratory. The time of permanence in ice was seemingly sufficient to cause partial freezing of blood into the syringe. When the sample was prepared for testing in the laboratory by thoroughly manual mixing, the frozen blood cells broke upon thawing, thus releasing a significant amount of potassium into the blood and finally leading to formulate a spurious diagnosis of hyperkalemia. Notably, thoughtful syringe mixing (and hence warming) by the technician before testing was probably sufficient to thawed the blood partially frozen, so that neither sample aspiration alerts or flagged results were generated by the analyser.

## Solution

To prevent freezing of heparinized blood due to direct contact of the syringe with ice used as coolant, whole blood samples for blood gas analysis should only be delivered to the laboratory at room temperature, within 30 minutes from collection.

## Discussion

Laboratory testing remains highly vulnerable to many preanalytical mistakes, and blood gas analysis makes no exception to this rule (*6*, *7*). Good quality laboratory practices, such as an accurate development of preanalytical activities and quality control procedures are essential for assuring that that in vitro test results actually reflect *in vivo* status. Recent statistics attests that the frequency of occult haemolysis is significant in whole blood samples referred to the laboratory for blood gas analysis, typically comprised between 4% and 13% (*8*, *9*). The assessment of these haemolysed specimens would then generate a number of spurious laboratory test results, not limited to potassium, but also involving pO_2_, SO_2_ and pCO_2_ (*10*).

According to the Clinical and Laboratory Standards Institute (CLSI) document GP43-A4, which is no longer available on the CLSI website, blood gas analysis samples which can be analysed within 30 minutes should be delivered to the laboratory at room temperature. When delayed analysis (i.e., > 30 minutes after blood drawing) may be unavoidable, the specimen should be immersed in coolant (but not ice), sufficiently large to allow immersing the entire barrel of the syringe (*11*). Although this was the reference procedure in place in our hospital at the time of this case report, the indications were not appropriately followed by the nurse, who entirely immersed the syringe into ice for approximately 15 minutes before being removed by the laboratory staff upon arrival in the laboratory. This relatively short time of direct contact with ice was seemingly sufficient to cause partial freezing of blood and thereby blood cell rupture upon thawing, thus promoting release of potassium and other intracellular substances into the blood (*12*). The increased value of pCO_2_, as well as the decreased values of pO_2_ and SO_2_ may have also been partially due to haemolysis, since variations of these three parameters alongside the same direction (*i.e.*, decrease of both pO_2_ and SO_2_ combined with increased pCO_2_) have been reported after experimental haemolysis of whole blood (*10*).

Unlike the former CLSI endorsements, the Croatian Society of Medical Biochemistry and Laboratory Medicine (CSMBLM) has recently published updated recommendations for preanalytical management of samples for blood gas analysis (*13*). Briefly, these samples should be analysed as soon as possible, not later than 30 minutes, whilst transportation in ice is strongly discouraged because plastic syringes appear more permeable to gases at lower temperatures and more vulnerable to spurious haemolysis. When the time between blood drawing and delivery to the laboratory is > 30 minutes, another sample shall be recollected.

In our case report, the overall increase of cell-free haemoglobin between the two specimens was approximately 0.45 g/L. Previous evidence was published that such a variation of the haemolysis index may be associated with a concomitant increase of potassium values comprised between 0.1 - 0.6 mmol/L (mean increase, 0.25 mmol/L), which does not completely justify the difference observed between the first and the second blood sample (*14*). Albeit it is hence conceivable that other biological or preanalytical factors may have contributed to increase the potassium concentration measured in the blood gas syringe, this does not attenuate the important evidence of a considerable increase of haemolysis in the first specimen. Notably, 15 minutes of direct contact with ice may be sufficient indeed to generate a significant degree of haemolysis in blood tubes or in blood gas syringes. As earlier demonstrated by Woolgar, maintaining a blood tube in a cooling bath (- 9 °C) for 10 minutes is sufficient to increase haemolysis up to 76% (*15*).

According to the evidence garnered from this case report, the CSMBLM recommendations for sample transportation appear more appropriate than those formerly issued by the CLSI, since they would actually prevent potential risks of haemolysis due to direct contact of syringes with ice used as coolant. This case also emphasizes the compelling need of developing a new generation of point of care analyser equipped with interference indices assessment. Finally, our data reinforce previous evidence on vagaries and inaccuracies in measurement of whole blood potassium, further underlining the importance to interpret potassium values according to clinical context and condition of patients for whom the test is performed (*16*).

## What YOU should/can do in your laboratory to prevent such errors

Whole blood samples for blood gas analysis should be delivered to the laboratory at room temperature, within 30 minutes from collection.
When the time between blood drawing and sample delivery the laboratory for whole blood gas analysis is > 30 minutes, another sample should be collected.
Potassium values shall always be interpreted according to clinical context and condition of patients for whom the test is performed.
